# High-flow nasal cannula oxygen therapy was effective for dysphagia associated with respiratory muscle paralysis due to cervical spinal cord injury: A case report

**DOI:** 10.1097/MD.0000000000026907

**Published:** 2021-08-13

**Authors:** Yoshihiro Watanabe, Toshiaki Tamura, Ryota Imai, Koki Maruyama, Mayumi Iizuka, Satomi Ohashi, Seigo Yamaguchi, Tatsunori Watanabe

**Affiliations:** a Department of Rehabilitation, Uonuma Kikan Hospital, Niigata Prefecture, Japan; b Department of Speech, Language, and Hearing Sciences, Niigata University of Health and Welfare, Niigata Prefecture, Japan; c Department of Emergency and Critical Care, Uonuma Kikan Hospital, Niigata Prefecture, Japan; d Department of Anesthesiology, Uonuma Kikan Hospital, Niigata Prefecture, Japan.

**Keywords:** dysphagia, high-flow nasal cannula oxygen therapy, low cervical spinal cord injury, swallowing rehabilitation

## Abstract

**Rationale:**

Respiratory muscle paralysis due to low cervical spinal cord injury (CSCI) can lead to dysphagia. Noninvasive positive airway pressure (PAP) therapy can effectively treat this type of dysphagia. High-flow nasal cannula (HFNC) oxygen therapy can generate a low level of positive airway pressure resembling PAP therapy, it may improve the dysphagia.

**Patient concerns:**

The patient was an 87-year-old man without preexisting dysphagia. He suffered a CSCI due to a dislocated C5/6 fracture, without brain injury, and underwent emergency surgery. Postoperatively (day 2), he complained of dysphagia, and the intervention was initiated.

**Diagnosis:**

Based on clinical findings, dysphagia in this case, may have arisen due to impaired coordination between breathing and swallowing, which typically occurs in patients with CSCI who have reduced forced vital capacity.

**Interventions:**

HFNC oxygen therapy was started immediately after the surgery, and swallowing rehabilitation was started on Day 2. Indirect therapy (without food) and direct therapy (with food) were applied in stages. HFNC oxygen therapy appeared to be effective because swallowing function temporarily decreased when the HFNC oxygen therapy was changed to nasal canula oxygen therapy.

**Outcomes:**

Swallowing function of the patient improved and he did not develop aspiration pneumonia.

**Lessons:**

HFNC oxygen therapy improved swallowing function in a patient with dysphagia associated with respiratory-muscle paralysis following a CSCI. It may have prolonged the apnea tolerance time during swallowing and may have improved the timing of swallowing. HFNC oxygen therapy can facilitate both indirect and direct early swallowing therapy to restore both swallowing and respiratory function.

## 1 Introduction

Respiratory muscle paralysis due to low cervical spinal cord injury (CSCI) and chronic obstructive pulmonary disease can lead to dysphagia caused by disrupted coordination between breathing and swallowing.^[1,2]^ Noninvasive positive airway pressure (PAP) therapy can effectively treat this type of dysphagia by improving coordination between breathing and swallowing.^[3,4]^ High-flow nasal cannula (HFNC) oxygen therapy generates a low level of positive airway pressure by administering a constant concentration of heated and humidified high flow oxygen.^[5]^ Thus, like PAP therapy, it may improve the discoordination between breathing and swallowing in patients with respiratory impairment. Here, we report a case in which HFNC oxygen therapy contributed to the improvement in swallowing in a patient with dysphagia due to respiratory muscle paralysis after low CSCI. This case report was approved by the ethics committee of the Uonuma Kikan Hospital (2021-3-002), and written informed consent was obtained from the patient.

## 2 Case

**Figure 1:**
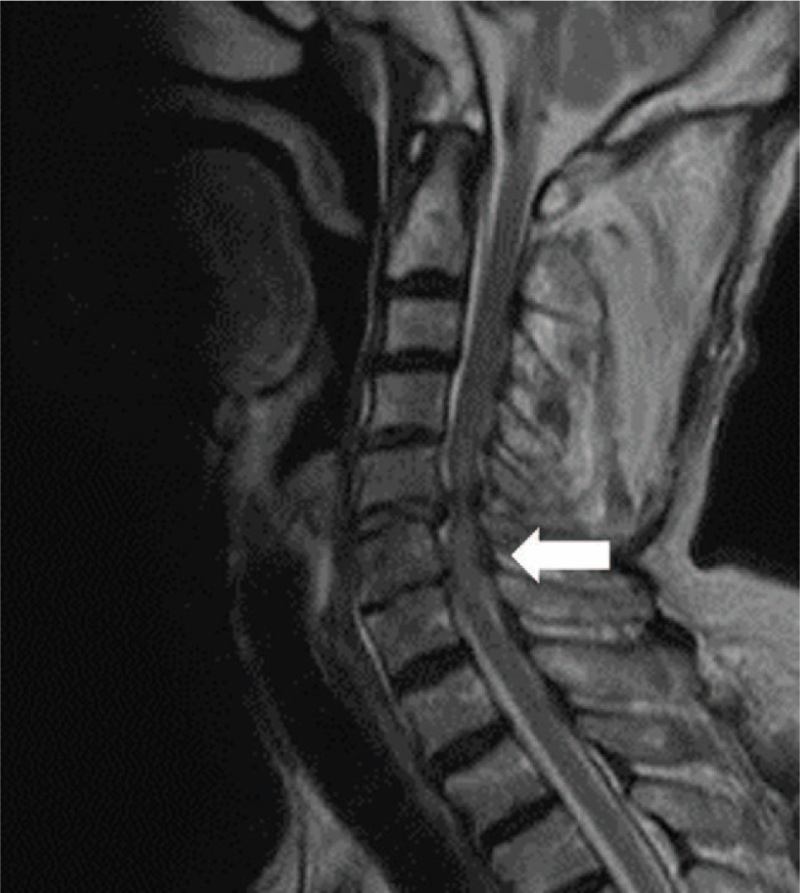
MRI (T2-weighted image). A high signal was found in the cervical spinal cord at the C5/6 level. MRI = magnetic resonance imaging.

The patient was an 87-year-old man (height 160 cm, weight 55.0 kg) with a medical history of hypertension and esophageal cancer. He had no history of dysphagia and had normal activities of daily living (ADL). He suffered CSCI due to a dislocated C5/6 fracture without brain injury (Fig. 1) and underwent emergency surgery involving posterior spinal fusion, laminoplasty, and laminectomy. Postoperatively (day 2), he complained of dysphagia, and intervention was initiated.

On examination, he was conscious without cognitive deficits. A manual muscle test showed 4/4 for the upper limbs, 0/0 for the lower limbs, and 0 for the trunk. He had no sensation in the upper or lower extremities and needed assistance with all ADL, namely, the Frankel classification ^[6]^ was A. His Barthel Index was 0/100. He had paralysis of the intercostal muscles and an abdominal paradox. An HFNC oxygen therapy (Optiflow; Fisher & Paykel Healthcare, Auckland, New Zealand) with FiO_2_ 0.4 and 40 L/min was used due to improved oxygenation, humidification, superior comfort, and patient tolerance.

His respiratory rate was 16 breaths/min, and percutaneous oxygen saturation (SpO_2_) was 98%. A mini-tracheostomy cannula (Mini-Trach II; Portex, Smiths Medical Japan Ltd., Tokyo, Japan) was implanted for sputum suction. This mini-tracheostomy cannula was usually closed during breathing and swallowing, and the cap was opened only when suctioning sputum. The patient was also equipped with a neck brace (Philadelphia Cervical Collar; Ossur, Reykjavik, Iceland). An assessment of swallowing function revealed no paralysis of the tongue or face. In the repetitive saliva swallowing test (RSST: normal is more than 3 times per 30 s),^[7]^ he swallowed six times in 30 s. However, he had wet hoarseness in 3 mL of thick water-swallowing test. Therefore, only indirect therapy (without food) was performed. In addition, his functional oral intake scale (FOIS)^[8]^ was Level 1, which means “No oral intake”.

**Figure 2:**
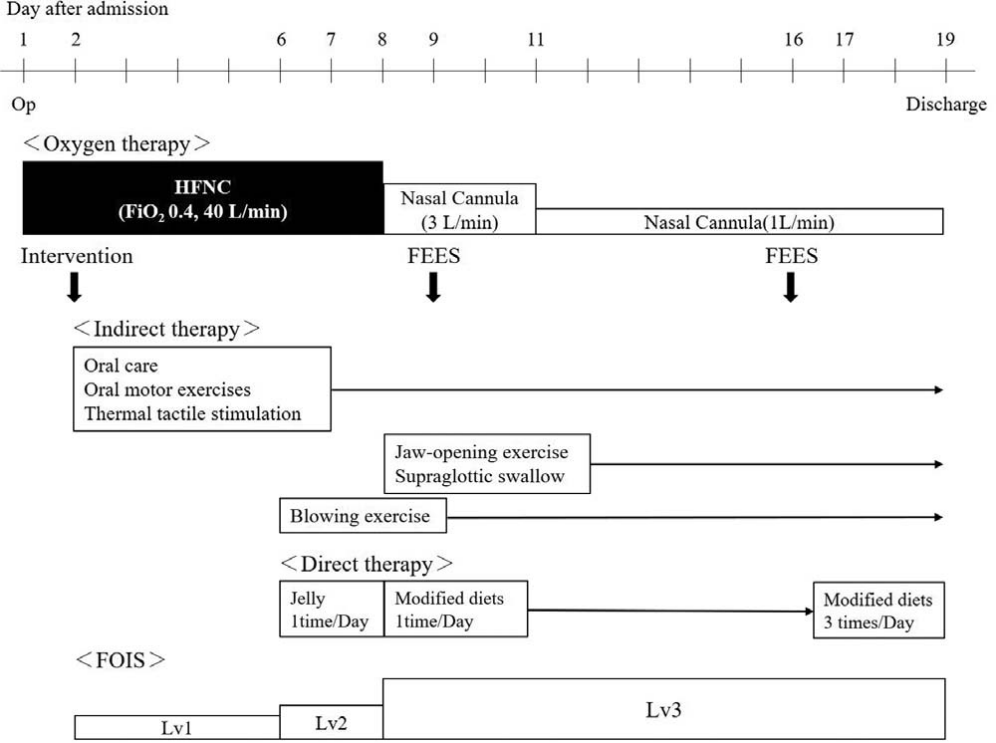
Clinical course. Oral intake and the course of swallowing rehabilitation in response to oxygen therapy.

The course of swallowing rehabilitation is shown in Fig. 2. With continued rehabilitation, wet hoarseness when swallowing 3 mL of thick water resolved on day 6, and direct therapy (with food) was initiated (FOIS: Lv2). Although the neck brace was temporarily removed during direct therapy, we carefully avoided neck extension. On day 7, respiratory, vocal, and swallowing functions were assessed with HFNC oxygen therapy (FiO_2_ 0.4, 40 L/min) and with nasal cannula oxygen therapy (3 L/min) to compare swallowing function between the different oxygen therapies. Posture was standardized to 60° bed-up, and evaluations were conducted within the same intervention time. Before evaluation, we confirmed that respiratory rate, SpO_2_, or other vital signs did not change due to the switch in oxygen therapy (Table 1). Regarding respiratory and vocal function, maximum expiration time (MET) and maximum phonation time (MPT) were measured, HFNC oxygen therapy made them longer than nasal cannula oxygen therapy. Regarding swallowing function, the RSST showed one more frequency with the nasal cannula oxygen therapy, but there was no significant difference between the two therapies. In both 3 ml Water-Swallowing Test (WST: Best is 5, Worst is 1)^[9]^ and the Food Test (FT: Best is 5, Worst is 1) with jelly,^[9]^ wet hoarseness and wheezing (3/5 points) were observed with nasal cannula oxygen therapy but not with HFNC oxygen therapy (5/5 points).

**Table 1:** Comparison of respiratory, vocal and swallowing function evaluation (Day 7).

	HFNC (40 L/min)	Nasal Cannula (3 L/min)
SpO_2_ (%)	97	98
RR (number of times per minute)	19	20
MET (s)	14	12
MPT (s)	12	8
RSST (number of times per 30 s)	7	8
WST (points)	5/5	3/5
FT (points)	5/5	3/5

**Figure 3:**
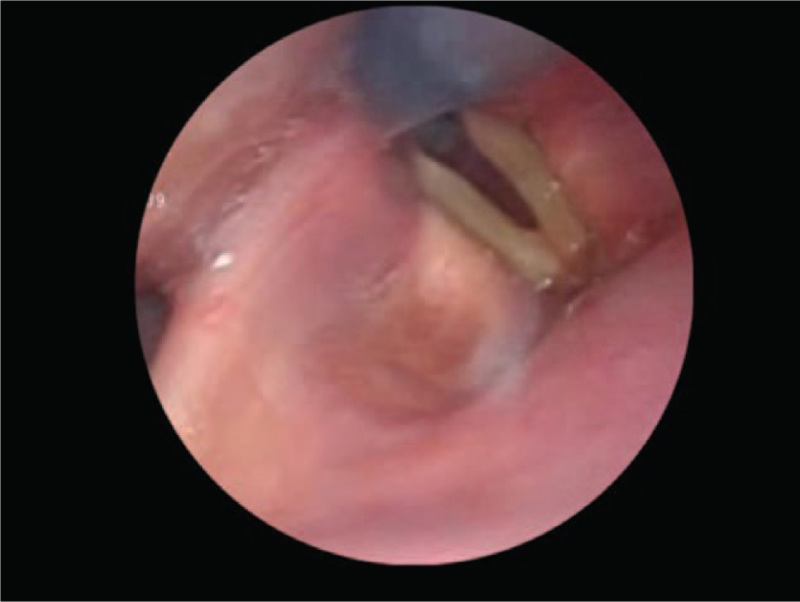
Flexible endoscopic evaluation of swallowing. The vocal cords opened early after swallowing, and the residual-colored water in the pharynx flowed into the vocal cords with inspiration.

On day 8, oxygen therapy was changed to nasal cannula oxygen therapy (3 L/min), and the patient was fed one meal a day of jelly porridge (FOIS: Lv3). On day 9, a flexible endoscopic evaluation of swallowing (FEES) was performed to evaluate swallowing function. FEES showed no paralysis of the laryngopharynx, soft palate elevation was good, and glottal closure was observed during speech and coughing. Moreover, the vocal cords opened early after swallowing, and the residual-colored water in the pharynx flowed into the vocal cords with inspiration (Fig. 3). Based on the FEES results, we encouraged multiple swallows and post-swallowing coughs during meals to reduce aspiration risk. The findings of FEES on day 16 improved compared to those on day 9. From day 17, the frequency of meals was increased to three per day. Day 19 swallowing function reassessment showed an overall improvement on the RSST (7 times/30 s) and a WST of 5/5 points despite a mini-tracheostomy cannula still in place. The patient was transferred to another hospital on day 19 for further rehabilitation. Postoperative aspiration pneumonia was not observed.

## 3 Discussion

HFNC oxygen therapy improved swallowing function in a patient who presented with dysphagia associated with respiratory muscle paralysis following CSCI. HFNC oxygen therapy can facilitate both indirect and direct early swallowing therapy to restore both swallowing and respiratory function. In this case, dysphagia was thought to be triggered by CSCI because there was no history of dysphagia prior to the injury. The C5/6 level CSCI was unlikely to be the direct cause since it did not damage the nerves innervating the muscle lifting the soft palate and larynx. However, dysphagia has been reported in a patient with reduced forced vital capacity (FVC) who suffered low CSCI.^[1]^ It was known that the FVC of patients with low CSCI, such as this patient, is significantly decreased in comparison to healthy individuals.^[10]^ Although we did not measure postoperative FVC, this patient's FVC was considered to be reduced because this patient had an abdominal paradox. Therefore, the decrease in FVC following CSCI was considered the primary etiological factor in the development of dysphagia in this case.

Dysphagia, in this case, may have arisen due to impaired coordination between breathing and swallowing, which typically occurs in patients with CSCI who have reduced FVC.^[1]^ In healthy individuals, breathing after swallowing is often resumed at expiration, which prevents aspiration of pharyngeal residue.^[11,12]^ However, it has been suggested that patients with respiratory-associated impaired coordination have a prolonged apnea duration during swallowing, and breathing during swallowing is more likely to be resumed at inspiration.^[3]^ In addition, patients with dysphagia have a longer swallowing latency, suggesting a delay in the timing of swallowing in the respiratory cycle.^[13]^ As a result, it has been concluded that breathing is more likely to be resumed with inspiration after swallowing.^[13]^ Therefore, these mechanisms may cause the laryngeal closure structure, such as the epiglottis and vocal cords, to open before the end of swallowing, making the patient susceptible to aspiration. Similarly, in this case, the FEES performed on day 9 showed that the vocal cords were opened early after swallowing and colored water flowed into the larynx with inspiration.

There are two possible mechanisms for the improvement in HFNC oxygen therapy. First, HFNC oxygen therapy may have prolonged the apnea tolerance time during swallowing as indicated by the prolonged MET and MPT than those with nasal cannula oxygen therapy on day 7. It suggests that HFNC oxygen therapy caused a temporary increase in lung volumes by positive end-expiratory pressure effect.^[14]^ As mentioned above, in the case of respiratory distress and a short apnea tolerance time, early resumption of breathing causes aspiration by opening the laryngeal closure structures prior to swallowing. However, prolonged apnea tolerance allows the laryngeal closure structure to remain closed until the end of swallowing, and aspiration is considered to have improved. This hypothesis may be supported by the fact that previous studies in healthy individuals have shown that HFNC oxygen therapy prolongs the laryngeal vestibule closure time during swallowing with increased oxygen flow.^[15]^ Second, HFNC oxygen therapy may have improved the timing of swallowing. The high-flow stimulation of HFNC oxygen therapy has the effect of shortening the latency of the swallowing reflex,^[16]^ apnea time during swallowing may be reduced, thereby reducing the risk of aspiration.

HFNC oxygen therapy could perform the enforcement of direct therapy in the early phase without any adverse events such as aspiration pneumonia, similar to previous cases.^[17]^ Since HFNC oxygen therapy is a device that delivers oxygen through the nasal cavity, appropriate assessment of respiratory and swallowing functions allows direct therapy to proceed incrementally, even since the beginning of HFNC oxygen therapy use. Thus, HFNC oxygen therapy can lead to early oral intake and shorter hospital stays because it prevents unnecessary periods of fasting and disuse of the swallowing function by promoting swallowing rehabilitation and improvement in dysphagia due to respiratory muscle paralysis.^[18]^ However, one limitation of our study is that it was difficult to perform FEES of the baseline because HFNC oxygen therapy was used immediately after surgery. In the future, it is necessary to objectively demonstrate the effects of HFNC oxygen therapy on swallowing function using FEES or a videofluoroscopic swallowing study.

In conclusion, HFNC oxygen therapy was effective in treating dysphagia in a patient with respiratory impairment due to CSCI. HFNC oxygen therapy may have a positive effect on swallowing function in patients with dysphagia related to respiratory muscle paralysis due to CSCI.

## Author contributions

**Investigation:** Yoshihiro Watanabe, Toshiaki Tamura, Ryota Imai, Koki Maruyama, Mayumi Iizuka, Satomi Ohashi, Seigo Yamaguchi, Tatsunori Watanabe.

**Methodology:** Yoshihiro Watanabe, Toshiaki Tamura, Ryota Imai, Koki Maruyama, Mayumi Iizuka, Satomi Ohashi, Seigo Yamaguchi, Tatsunori Watanabe.

**Project administration:** Yoshihiro Watanabe, Toshiaki Tamura, Ryota Imai, Koki Maruyama, Mayumi Iizuka, Satomi Ohashi, Seigo Yamaguchi, Tatsunori Watanabe.

**Supervision:** Tatsunori Watanabe.

**Validation:** Yoshihiro Watanabe, Toshiaki Tamura, Ryota Imai, Koki Maruyama, Mayumi Iizuka, Satomi Ohashi, Seigo Yamaguchi, Tatsunori Watanabe.

**Writing – original draft:** Yoshihiro Watanabe.

**Writing – review & editing:** Yoshihiro Watanabe, Toshiaki Tamura, Ryota Imai, Koki Maruyama, Mayumi Iizuka, Satomi Ohashi, Seigo Yamaguchi, Tatsunori Watanabe.
